# On the History of Tuberculous Meningitis

**Published:** 1840-05-11

**Authors:** W. W. Gerhard


### MEDICAL EXAMINER.

DEVOTED TO MEDICINE, SURGERY, AND THE COLLATERAL SCIENCES.

No. 19.] PHILADELPHIA, SATURDAY, MAY 11, 1840. [Vol. III.

### ON THE HISTORY OF TUBERCULOUS MENINGITIS.

BY W. W. GERHARD, M. D.

In the last number of the Medical Examiner I published a translation of a portion of the article upon Meningitis, by M. Guersant, and the correspondence which afterwards was published in the Archives, relative to this subject. In itself, the matter was of little importance, but as the discovery of the true nature of the disease did not arise from accident, but was the direct consequence of a course of laborious investigation, I attach more importance to the matter than I should do under other circumstances.

In the article upon Meningitis by M. Guersant, the attempt is made to refer the discovery of the connexion between tubercles in the membranes of the brain and acute hydrocephalus, as far back as the time of Willis. This is about as reasonable as it would be to ascribe all possible researches to the older writers; the germ of every thing that has been discovered, and probably of every thing that will be discovered, is to be found in the ancient writers, but the mere obscure allusion does not constitute positive knowledge. In various writings, the description of tubercles in the brain and its membranes may be found, but they are invariably stated to be mere complications, and usually accidental ones of acute hydrocephalus. The accounts of writers are singularly uniform on this point, differing only in this respect, that one class represents the tuberculous diathesis as a predisposing cause of the disease, while another attributes the inflammation to the tubercles as the immediate exciting cause. Both of these suppositions are to a certain extent well founded, the former much more frequently than the latter.

The last, and by far the most complete account of the tuberculous granulations as occurring in acute hydrocephalus, is to be found in the memoir of Dance on this subject, the concluding part of this paper appeared in the Archives in 1830, a little more than two years before the commencement of the researches of Dr. Rufz and myself. M. Dance has given a correct though incomplete description of the tuberculous granulations in the pia mater, which are, as he states, a frequent cause of acute hydrocephalus. He describes the granulations as a cause of the disease, and in fact recognises the granulations themselves only in those cases in which they have attained a considerable size, and are evidently tuberculous. In the greater part of the cases which he describes, the disease was certainly the ordinary tuberculous meningitis, but as the tuberculous granulations were small, and, comparatively speaking, concealed by the effusion of serum and lymph, they must have escaped attention. M. Dance expressly stated that these granulations were merely a complication of the disease, and gives the following definition of acute hydrocephalus. It is "an inflammation seated primarily or secondarily in the cerebral ventricles, (in their internal membrane and the layer of subjacent cerebral substance,) giving rise to a more or less considerable serous effusion into these cavities, afterwards to a more or less extended softening of these walls, complicated frequently with meningitis, especially at the base of the brain, and producing a form of symptoms differing in general from those of all other cerebral diseases, which is owing in great part to its peculiar position, and, above all, to the *effusion* produced by it. On this account we believe that it is proper to preserve the term acute hydrocephalus." (Archives, vol. 22, p. 322.)

The tuberculous or scrofulous diathesis which M. Dance, in common with many others, noticed as complications of acute hydrocephalus, is, in fact, much more important than it seemed to him. The lymphatic temperament is the ultimate cause of the disease. According to the opinion of Mons. D., it acts in two ways. In the one, tubercles are already present in the membranes of the brain, and "foment," to use the expression of M. Dance, the inflammation. This is the variety of the disease in which the tuberculous character of the disease was so evident that there could be no doubt as to its real nature. The second variety is that in which the lymphatic temperament is the remote cause of the disorder; this is also generally admitted by many of the older writers, not merely a cause favouring the development of hydrocephalus.

I have confined my remarks to the works of M. Dance, as he is the only writer whose views approach nearly to our own, and by a reference to his article it will be seen that this approximation is limited entirely to one thing—a more accurate anatomical description of tubercles when they are evident in the pia mater than was given by preceding writers. He has totally passed over the cases in which the tuberculous disease must have existed, although, as yet, in a forming state, and he has not hinted at the pathology of the most frequent form of the disease. This is that in which the tuberculous granulations are secreted by the same inflammatory process which constitutes the disease, the scrofulous temperament acting merely as a remote cause.

I will next inquire in what the observations of M. Rufz and myself differ from them. It is in this—that we state and prove, by rigid deductions, that the acute meningitis, or acute hydrocephalus, if the older term be preserved, is really a tuberculous disease, it is inflammatory in its direct action, and the tuberculous granulations are either formed already in the brain and followed by the acute inflammation, or they are secreted by the inflammatory process, the vessels throwing out at the same time lymph and tuberculous matter. This disease is by far the most frequent of the acute inflammatory disorders of the brain in children, especially from the age of two years to that of puberty, and we therefore separate this disease from other acute affections of the brain, laying stress upon the peculiar nature of the disease as connected with the tuberculous secretion, and looking upon the affection as one and the same whether the inflammation attacks the membranes only, or extends to the substance of the brain, and whether there is or is not an effusion of serum into the ventricles. This view then of the pathology of the disease renders the term hydrocephalus obviously improper, and it was this which induced us to substitute that of tuberculous meningitis, which has since come into general use.

The new view of the disease was a necessary consequence of the improved condition of pathological knowledge, and of the methods of examining and of drawing deductions from facts, and it would necessarily have been taken by any one who had followed out the same course of research. That it was not adopted by others previously to the observations of M. Rufz and myself, is very obvious from the examination of the essay by M. Dance, which was published but little more than two years before the commencement of our researches. From the year 1830 to 1832, but little attention seemed directed to this subject, and no memoir in reference to it appeared ; in the months of April and May of that year the cholera appeared at Paris; after its cessation, I ceased to follow the practice of the larger hospitals, and commenced a series of studies at the children's hospital at the beginning of June, 1832 ; after observing at the hospital for some weeks, I determined to continue my observations there for as long a period as practicable, and was constantly engaged in noting cases and studying the diseases of children until January, 1834, a period of eighteen months. Soon after the commencement of these researches, M. Rufz, who was attached to the service of M. Guersant, associated himself with me ; to a certain extent, we were each studying the diseases in different wards of the hospital, but agreed to compare together our notes, and thus arrive at some conclusions more rapidly than we otherwise should do. The kindness of MM. Jadelot and Bouneau enabled me to pursue these observations during this period, and the position of Dr. Rufz at the hospital gave him the necessary facilities for observing the patients under the care of Drs. Guersant and Baudelocque. We were very soon struck with the tuberculous granulations which were so evident in some cases of meningitis ; in other cases we did not at first venture to regard these granulations as tuberculous, but we commenced a careful examination of the other organs of the body, and ascertained that in every case of a certain class, there was direct evidence of a tuberculous disease. From this circumstance and the examination of the brain, we finally arrived at the conclusion that the disease was strictly tuberculous. The disease was not separated by us from ordinary cases of meningitis, solely because it was connected with a tuberculous deposit; it presented, in most cases, a train of symptoms which are to a great degree peculiar, and have an individual character; although the distinctive signs of the disorder were not discovered until the anatomical characters had been ascertained.

The first publication made by either of us, was by Dr. Rufz, in February, 1833, in which he described the granulations, but did not yet connect them with the tuberculous diseases. This connection was ascertained by us in the course of the spring or summer of the same year, when we had collected a sufficient number of observations to justify us, as we thought, in drawing some conclusions from them. All the observations which I had noted, agreed in giving the same result, beginning from June, 1832: the later cases of Dr. Rufz coincided perfectly with them, but those noted previously to the months of September or October, did not lead to any definite result, because they were too imperfect. The earlier cases were incomplete, because Dr. Rufz did not then, at first, intend to continue his researches at the children's hospital beyond a short period, and therefore devoted less time to these notes than he afterwards gave to them. The first series of cases which demonstrated the nature of the disease were those which I had noted, but as the studies of both M. Rufz and myself were made afterwards in such a way as materially to assist our researches, the additions made to our knowledge of acute hydrocephalus, was regarded by us as the fruits of our joint researches.

The first publication which I made upon the subject was in the American Journal for February, 1834, in a paper written at Paris in September or October, 1833 ; the character of the disease is there stated. The second part of the memoir was published in May of the same year.

Dr. Rufz published his thesis on the same subject in 1835. These papers all related to acute hydrocephalus, or rather tuberculous meningitis, as it occurs in children, but in the year 1835, in the same journal, I published a memoir upon the acute hydrocephalus of adults, proving that it was identical with the tuberculous meningitis of children, and depended upon the same causes. This was the first memoir which proved that the disease was as distinctly characterized in adults as in children, and that the anatomical lesion was in both cases independent of the effusion of serum into the ventricles of the brain.

These remarks show that the disease was not described in such a manner as to give it a separate existence, until the researches of Dr. Rufz and myself. Since that period, many others, especially of the internes at the Children's Hospital, have written upon the same disease, which they agree in considering as a tuberculous meningitis. Amongst these later writers is the late M. Constant, who began his researches at the Children's Hospital in January, 1833, after M. Rufz and myself had collected a large proportion of cases upon the same subject. His observations were made less elaborately than those which led us to these conclusions, chiefly during a short visit in each morning; they, however, confirmed our previous researches. M. Guersant was in error when he seems to intimate that the researches of M. Constant upon this subject were anterior to ours. He had no knowledge of the intimate connection between the tuberculous diseases and acute hydrocephalus until they were pointed out to him at the hospital. Of the occasional connexion of tubercles of the membranes and meningitis he was no doubt aware, like all others who were acquainted with the previous publication upon this subject; but the knowledge of the true relations of the disease could scarcely have been attained without a course of laborious researches similar to those undertaken by us. The labour that we incurred is the chief reason why we attach importance to the subject.

